# Supplementary material for: Partial Biodegradable Blend for Fused Filament Fabrication: In-Process Thermal and Post-Printing Moisture Resistance
Source: Polymers (Basel). 2022 Apr 9;14(8):1527. doi: 10.3390/polym14081527 (PMC9025397; doi:10.3390/polym14081527)
Supplement: Supplementary file 1 [file polymers-14-01527-s001.zip › polymers-1671557-supplementary.pdf]

polymers

MDPI

Supplementary File S1: Minitab analysis for hydrolytic degradation (water absorption)

Partial biodegradable blend for fused filament fabrication: In-process thermal and post-printing moisture resistance

Muhammad Harris [1,2,*], Hammad Mohsin [3], Rakhshanda Naveed [4], Johan Potgieter [1], Kashif Ishfaq [4], Sudip Ray [5], Marie-Joo Le Guen [6], Richard Archer [7] and Khalid Mahmood Arif [8]

1 Massey Agrifood Digital Lab, Massey University, Palmerston North 4410, New Zealand; j.potgieter@massey.ac.nz
2 Industrial and Manufacturing Engineering Department, Rachna College of Engineering and Technology, Gujranwala 52250, Pakistan; engr.harris@.uet.edu.pk
3 Department of Polymer Engineering, National Textile University, Faisalabad 37610, Pakistan; mhammad@ntu.edu.pk
4 Industrial and Manufacturing Engineering Department, University of Engineering and Technology, Lahore 54890, Pakistan; rakhshanda@uet.edu.pk (R.N.); kashif.ishfaq@uet.edu.pk (K.I.)
5 New Zealand Institute for Minerals to Materials Research, Greymouth 7805, New Zealand; s.ray@auckland.ac.nz
6 Scion, Rotorua 3046, New Zealand; mariejoo.leguen@scionresearch.com
7 School of Food and Advanced Technology, Massey University, Palmerston North 4410, New Zealand; r.h.archer@massey.ac.nz
8 Department of Mechanical and Electrical Engineering, SF&AT, Massey University, Auckland 0632, New Zealand; k.arif@massey.ac.nz
* Correspondence: m.harris@massey.ac.nz

Citation: Harris, M.; Mohsin, H.; Naveed, R.; Potgieter, J.; Ishfaq, K.; Ray, S.; Guen, M.-J.L.; Archer, R.; Arif, K.M. Partial Biodegradable Blend for Fused Filament Fabrication: In-Process Thermal and Post-Printing Moisture Resistance. *Polymers* **2022**, *14*, 1527. https://doi.org/10.3390/polym14081527

Academic Editors: Roland Kuen Ren Chen and Yancheng Wang

Received: 23 March 2022
Accepted: 7 April 2022
Published: 9 April 2022

Publisher's Note: MDPI stays neutral with regard to jurisdictional claims in published maps and institutional affiliations.

Copyright: © 2022 by the authors. Submitted for possible open access publication under the terms and conditions of the Creative Commons Attribution (CC BY) license (https://creativecommons.org/licenses/by/4.0/).

The randomized multi-level general full factorial design of experiment (DoE) of the 1St trial for the effects of water absorption and in-process printing temperatures on tensile strength is provided in Table S1.

Table S1. DoE for 1st ANOVA analysis of water absorbed samples.

RunOrder	PtType	Blocks	Bed temperature	Printing temperature	Moisture Treatment	Tensile Strength
1	1	1	25	171	Treated	43.00672
2	1	1	55	171	Non-treated	40.01403
3	1	1	55	166	Non-treated	37.71559
4	1	1	25	166	Non-treated	36.12446
5	1	1	85	166	Treated	40.15167
6	1	1	85	171	Non-treated	43.10712
7	1	1	55	171	Treated	46.4119
8	1	1	25	161	Treated	39.565995
9	1	1	25	171	Non-treated	43.37669
10	1	1	55	166	Treated	44.06397
11	1	1	25	166	Treated	37.79949
12	1	1	85	161	Treated	42.2128
13	1	1	55	161	Treated	44.06397
14	1	1	55	161	Non-treated	42.99
15	1	1	85	171	Treated	79.49829
16	1	1	85	161	Non-treated	44.959735
17	1	1	25	161	Non-treated	38.92701
18	1	1	85	166	Non-treated	32.49289

WORKSHEET 1

General Factorial Regression: Tensile Strength versus Bed temperature, Printing temperature, Moisture Treatment

Factor Information

Factor	Levels	Values
Bed temperature	3	25, 55, 85
Printing temperature	3	161, 166, 171
Moisture Treatment	2	Non-treated, Treated

Analysis of Variance

Source	DF	Adj SS	Adj MS	F-Value	P-Value
Model	13	1347.1	103.63	1.59	0.350
Linear	5	726.8	145.37	2.23	0.229
Bed temperature	2	161.8	80.88	1.24	0.381
Printing temperature	2	384.2	192.08	2.94	0.164

Moisture Treatment	1	180.9	180.93	2.77	0.171
2-Way Interactions	8	620.3	77.54	1.19	0.464
Bed temperature*Printing temperature	4	324.3	81.08	1.24	0.419
Bed temperature*Moisture Treatment	2	135.9	67.93	1.04	0.433
Printing temperature*Moisture Treatment	2	160.1	80.07	1.23	0.384
Error	4	261.2	65.30		
Total	17	1608.4			

Model Summary

S	R-sq	R-sq(adj)	R-sq(pred)
8.08094	83.76%	30.98%	0.00%

Coefficients

Term	Coef	SE Coef	T-Value	P-Value	VIF
Constant	43.14	1.90	22.65	0.000	
Bed temperature					
25	-3.34	2.69	-1.24	0.283	1.33
55	-0.59	2.69	-0.22	0.836	1.33
Printing temperature					
161	-1.02	2.69	-0.38	0.725	1.33
166	-5.08	2.69	-1.89	0.132	1.33
Moisture Treatment					
Non-treated	-3.17	1.90	-1.66	0.171	1.00
Bed temperature*Printing temperature					
25 161	0.46	3.81	0.12	0.909	1.78
25 166	2.24	3.81	0.59	0.588	1.78
55 161	2.00	3.81	0.53	0.627	1.78
55 166	3.43	3.81	0.90	0.419	1.78
Bed temperature*Moisture Treatment					
25 Non-treated	2.85	2.69	1.06	0.350	1.33
55 Non-treated	0.87	2.69	0.32	0.764	1.33
Printing temperature*Moisture Treatment					
161 Non-treated	3.34	2.69	1.24	0.282	1.33
166 Non-treated	0.56	2.69	0.21	0.846	1.33

Regression Equation

Tensile Strength = 43.14 - 3.34 Bed temperature_25 - 0.59 Bed temperature_55
+ 3.93 Bed temperature_85 - 1.02 Printing temperature_161
- 5.08 Printing temperature_166 + 6.10 Printing temperature_171
- 3.17 Moisture Treatment_Non-treated
+ 3.17 Moisture Treatment_Treated
+ 0.46 Bed temperature*Printing temperature_25 161
+ 2.24 Bed temperature*Printing temperature_25 166
- 2.71 Bed temperature*Printing temperature_25 171
+ 2.00 Bed temperature*Printing temperature_55 161
+ 3.43 Bed temperature*Printing temperature_55 166
- 5.43 Bed temperature*Printing temperature_55 171
- 2.47 Bed temperature*Printing temperature_85 161
- 5.67 Bed temperature*Printing temperature_85 166
+ 8.13 Bed temperature*Printing temperature_85 171
+ 2.85 Bed temperature*Moisture Treatment_25 Non-treated
- 2.85 Bed temperature*Moisture Treatment_25 Treated
+ 0.87 Bed temperature*Moisture Treatment_55 Non-treated

- 0.87 Bed temperature*Moisture Treatment_55 Treated
- 3.71 Bed temperature*Moisture Treatment_85 Non-treated
+ 3.71 Bed temperature*Moisture Treatment_85 Treated
+ 3.34 Printing temperature*Moisture Treatment_161 Non-treated
- 3.34 Printing temperature*Moisture Treatment_161 Treated
+ 0.56 Printing temperature*Moisture Treatment_166 Non-treated
- 0.56 Printing temperature*Moisture Treatment_166 Treated
- 3.90 Printing temperature*Moisture Treatment_171 Non-treated
+ 3.90 Printing temperature*Moisture Treatment_171 Treated

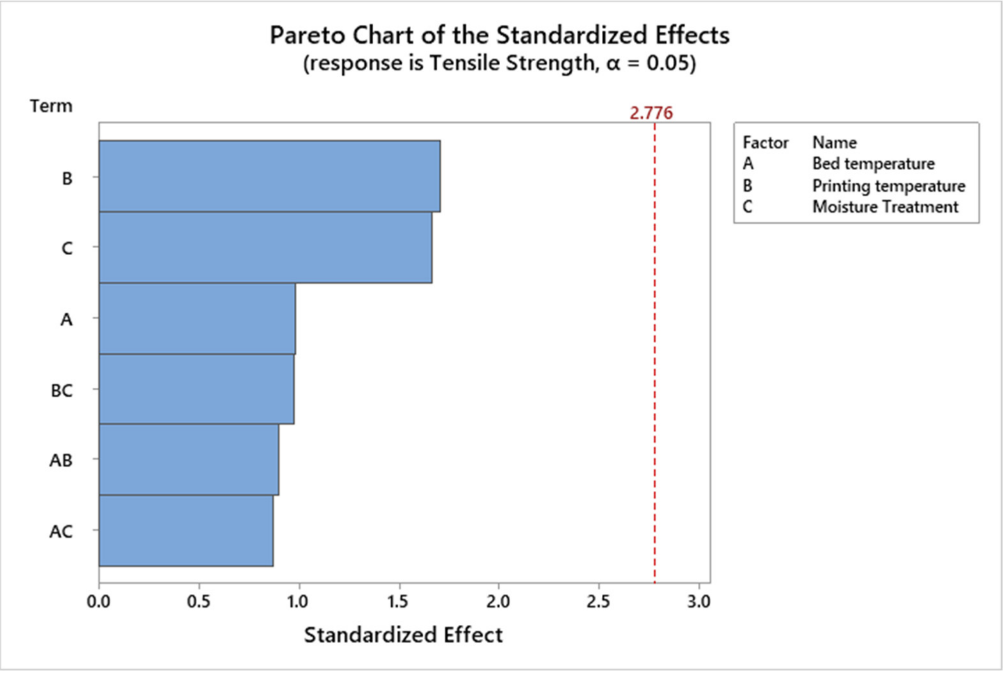

Figure S1. Pareto chart for the first trial of moisture treatment samples.

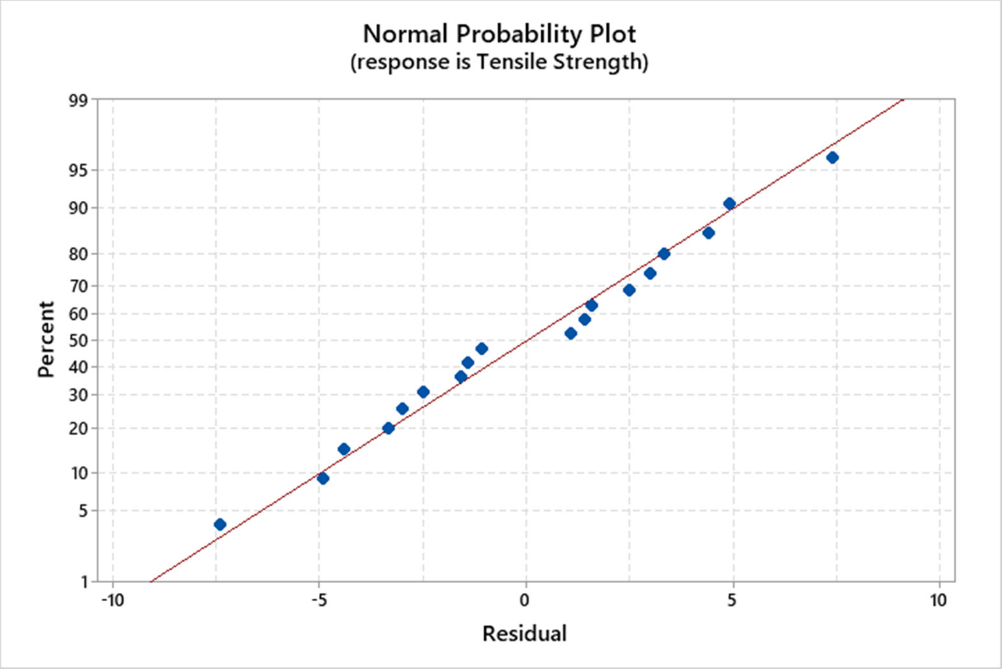

Figure S2. Normal probability plot for the first trial of moisture treatment samples.

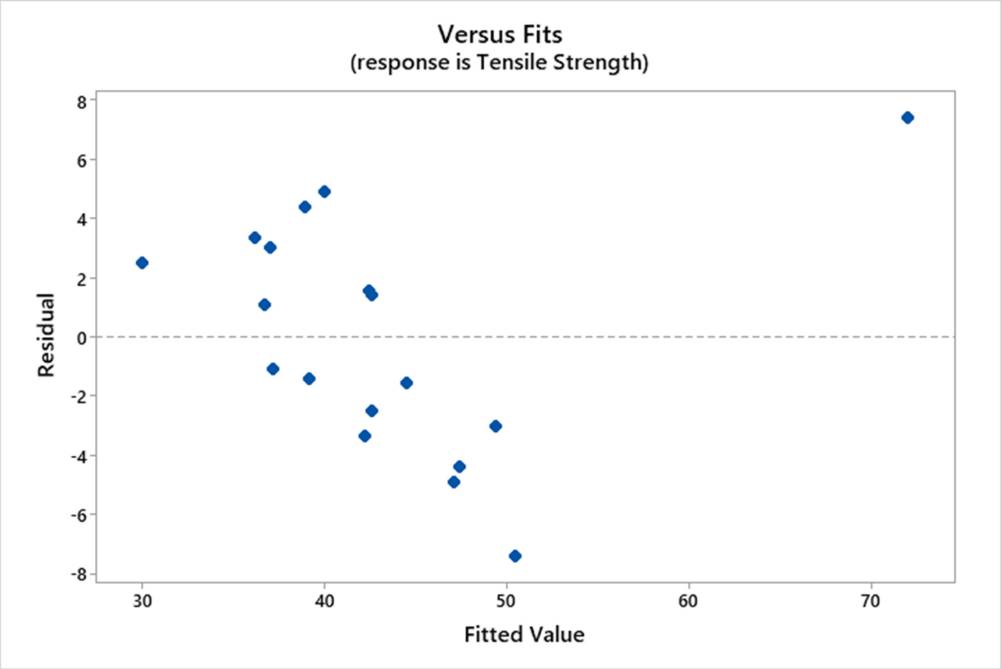

Figure S3. Versus fit plot for the first trial of moisture treatment samples.

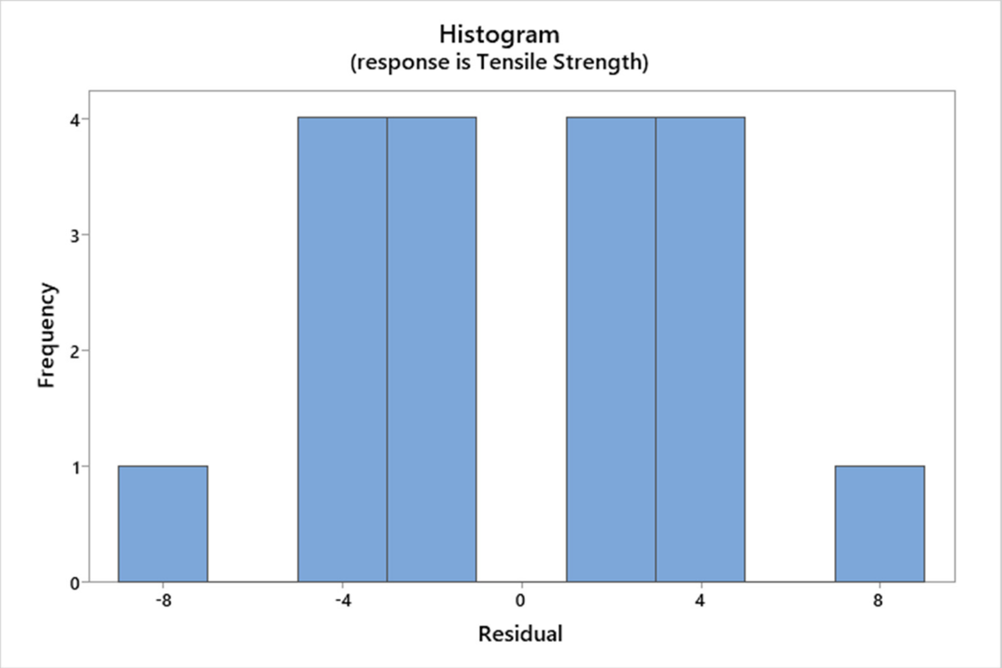

Figure S4. Histogram for the first trial of moisture treatment samples.

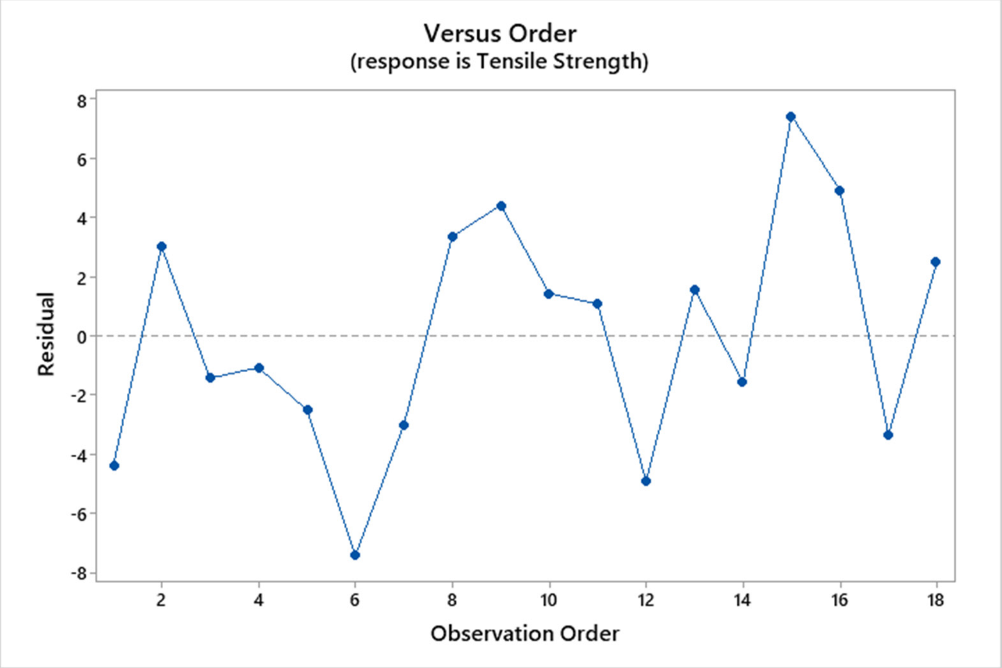

Figure S5. Versus order plot for the first trial of moisture treatment samples.

Corrected Moisture analysis excluding outlier (79 MPa)

The randomized multi-level general full factorial design of experiment (DoE) of 2nd trial for the effects of water absorption and in-process printing temperatures on tensile strength is provided in Table S2.

Table S2. DoE for 2nd ANOVA analysis of water absorbed samples.

RunOrder	PtType	Blocks	Bed temperature	Printing temperature	Moisture Treatment	Tensile Strength
1	1	1	25	171	Treated	43.00672
2	1	1	55	171	Non-treated	40.01403
3	1	1	55	166	Non-treated	37.71559
4	1	1	25	166	Non-treated	36.12446
5	1	1	85	166	Treated	40.15167
6	1	1	85	171	Non-treated	43.10712
7	1	1	55	171	Treated	46.4119
8	1	1	25	161	Treated	39.565995
9	1	1	25	171	Non-treated	43.37669
10	1	1	55	166	Treated	44.06397
11	1	1	25	166	Treated	37.79949
12	1	1	85	161	Treated	43
13	1	1	55	161	Treated	44.06397
14	1	1	55	161	Non-treated	42.99
15	1	1	85	171	Treated	50.3
16	1	1	85	161	Non-treated	44.9
17	1	1	25	161	Non-treated	38.92701
18	1	1	85	166	Non-treated	32.49289

As it is noted in the first trial of Minitab analysis, the moisture treatment appears with an outlier associated with treated (171 C, 85 C). The outlier is highlighted in versus fit plot. Furthermore, the histogram does not show the data to be normally distributed about the mean. Therefore, the analysis is re-performed by replacing the excluded outlier (79 MPa) with the new average of tensile strength at treated (171 C, 85 C). The analysis is as follow.

WORKSHEET 1

General Factorial Regression: Tensile Strength versus Bed tempearture, Printing temperature, Moisture Treatment

Factor Information

Factor	Levels	Values
Bed temperature	3	25, 55, 85
Printing temperature	3	161, 166, 171
Moisture Treatment	2	Non-treated, Treated

Analysis of Variance

Source	DF	Adj SS	Adj MS	F-Value	P-Value
Model	13	276.35	21.258	5.62	0.054
Linear	5	197.43	39.487	10.43	0.021
Bed temperature	2	27.90	13.949	3.69	0.124
Printing temperature	2	123.72	61.862	16.35	0.012
Moisture Treatment	1	45.81	45.811	12.11	0.025
2-Way Interactions	8	78.92	9.865	2.61	0.185
Bed temperature*Printing temperature	4	40.00	9.999	2.64	0.185
Bed temperature*Moisture Treatment	2	14.61	7.305	1.93	0.259
Printing temperature*Moisture Treatment	2	24.31	12.157	3.21	0.147
Error	4	15.14	3.784		
Total	17	291.49			

Model Summary

S	R-sq	R-sq(adj)	R-sq(pred)
1.94529	94.81%	77.93%	0.00%

Coefficients

Term	Coef	SE Coef	T-Value	P-Value	VIF
Constant	41.556	0.459	90.63	0.000	
Bed temperature					
25	-1.756	0.648	-2.71	0.054	1.33
55	0.987	0.648	1.52	0.203	1.33
Printing temperature					
161	0.685	0.648	1.06	0.350	1.33
166	-3.498	0.648	-5.39	0.006	1.33
Moisture Treatment					

Non-treated	-1.595	0.459	-3.48	0.025	1.00
Bed temperature*Printing temperature					
25 161	-1.239	0.917	-1.35	0.248	1.78
25 166	0.660	0.917	0.72	0.511	1.78
55 161	0.299	0.917	0.33	0.761	1.78
55 166	1.845	0.917	2.01	0.115	1.78
Bed temperature*Moisture Treatment					
25 Non-treated	1.271	0.648	1.96	0.121	1.33
55 Non-treated	-0.708	0.648	-1.09	0.336	1.33
Printing temperature*Moisture Treatment					
161 Non-treated	1.627	0.648	2.51	0.066	1.33
166 Non-treated	-1.018	0.648	-1.57	0.191	1.33

Regression Equation

Tensile Strength = 41.556 - 1.756 Bed tempearture_25 + 0.987 Bed tempearture_55
+ 0.769 Bed tempearture_85 + 0.685 Printing temperature_161
- 3.498 Printing temperature_166 + 2.813 Printing temperature_171
- 1.595 Moisture Treatment_Non-treated + 1.595 Moisture Treatment_Treated
- 1.239 Bed temperature*Printing temperature_25 161
+ 0.660 Bed temperature*Printing temperature_25 166
+ 0.578 Bed temperature*Printing temperature_25 171
+ 0.299 Bed temperature*Printing temperature_55 161
+ 1.845 Bed temperature*Printing temperature_55 166
- 2.143 Bed temperature*Printing temperature_55 171
+ 0.940 Bed temperature*Printing temperature_85 161
- 2.505 Bed temperature*Printing temperature_85 166
+ 1.565 Bed temperature*Printing temperature_85 171
+ 1.271 Bed temperature*Moisture Treatment_25 Non-treated
- 1.271 Bed temperature*Moisture Treatment_25 Treated
- 0.708 Bed temperature*Moisture Treatment_55 Non-treated
+ 0.708 Bed temperature*Moisture Treatment_55 Treated
- 0.563 Bed temperature*Moisture Treatment_85 Non-treated
+ 0.563 Bed temperature*Moisture Treatment_85 Treated
+ 1.627 Printing temperature*Moisture Treatment_161 Non-treated
- 1.627 Printing temperature*Moisture Treatment_161 Treated
- 1.018 Printing temperature*Moisture Treatment_166 Non-treated
+ 1.018 Printing temperature*Moisture Treatment_166 Treated
- 0.608 Printing temperature*Moisture Treatment_171 Non-treated
+ 0.608 Printing temperature*Moisture Treatment_171 Treated

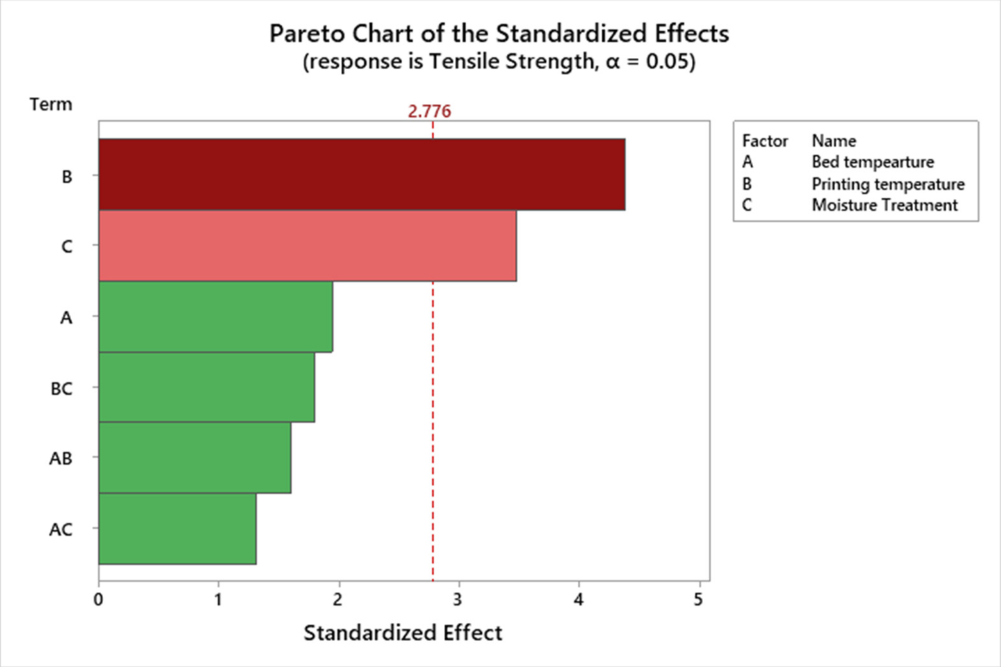

Figure S6. Pareto chart for corrected trial of moisture treatment samples
.

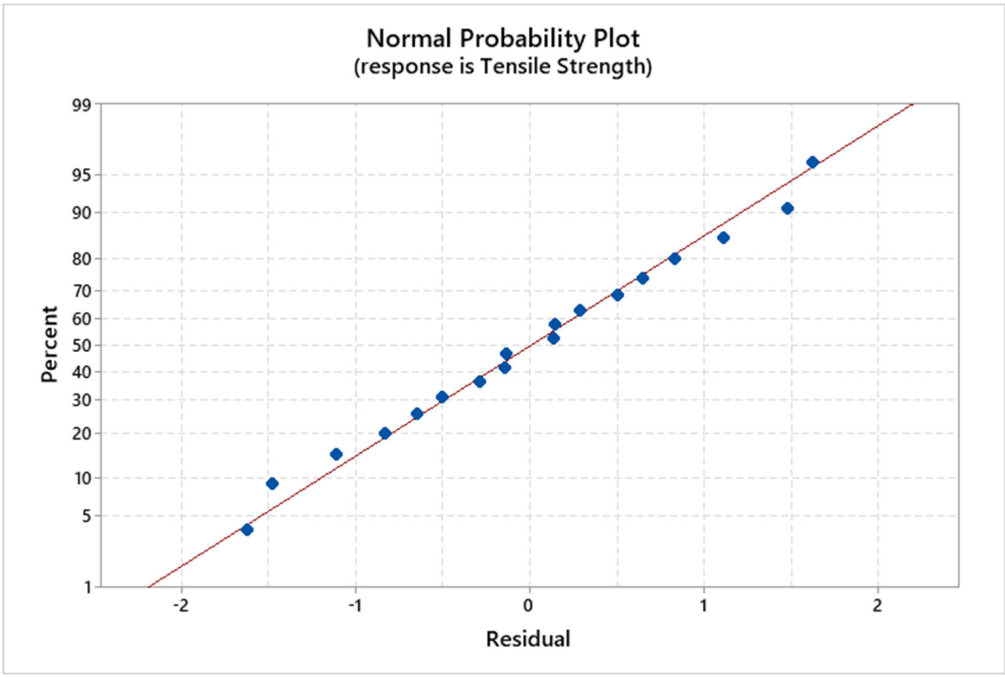

Figure S7. Normal probability plot for the corrected trial of moisture treatment samples.

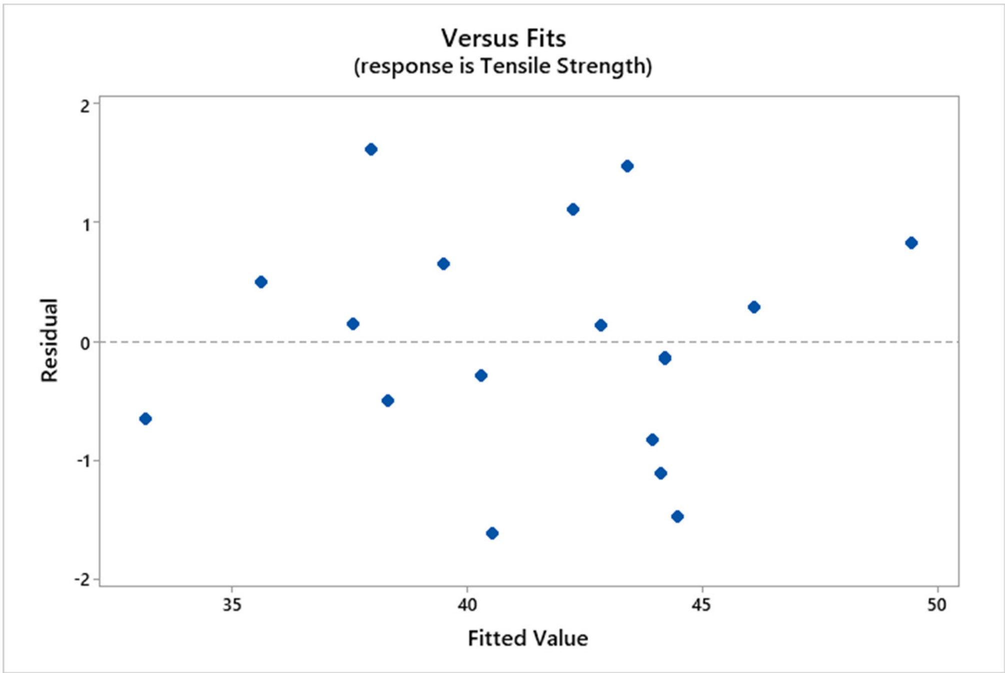

Figure S8. Versus fit plot for the corrected trial of moisture treatment samples.

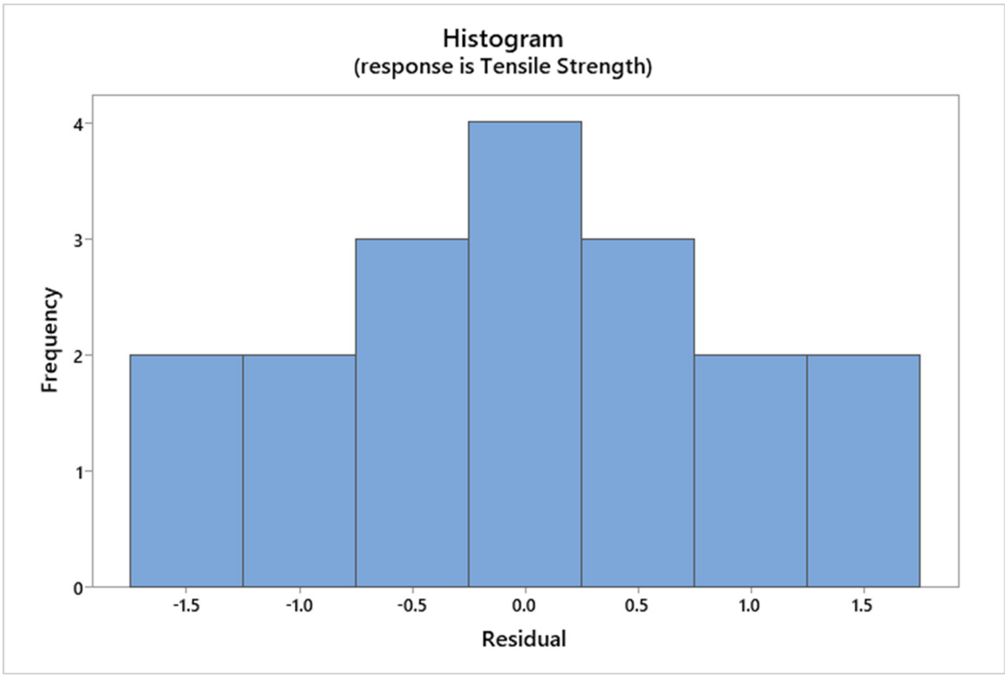

Figure S9. Histogram for the corrected trial of moisture treatment samples.

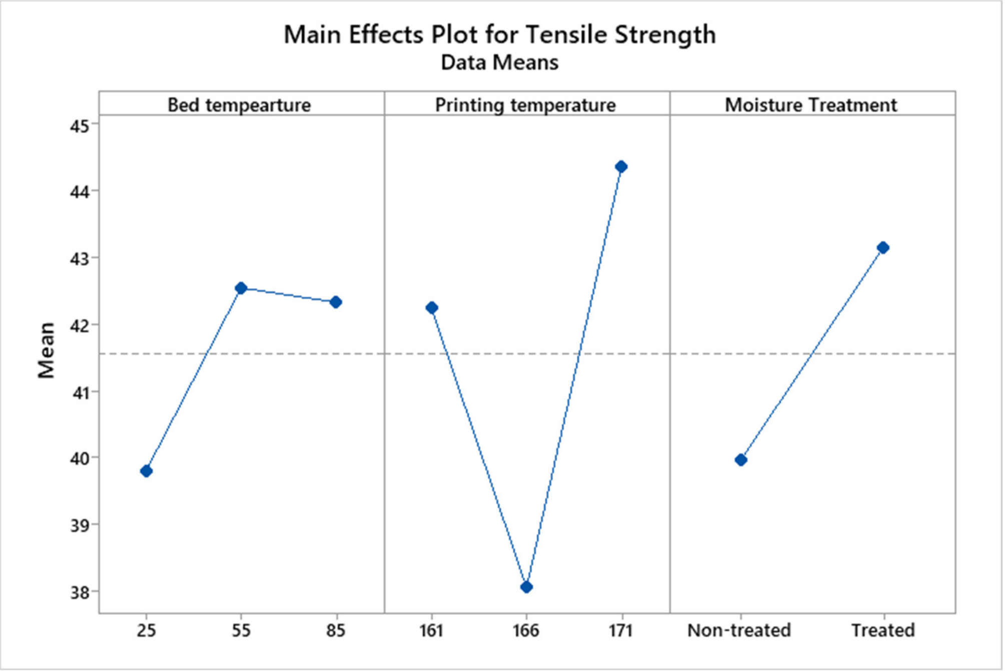

Figure S10. Main effects plot for corrected moisture treatment samples.
